# Porous Metal–Organic Frameworks for Light Hydrocarbon Separation

**DOI:** 10.3390/molecules28176337

**Published:** 2023-08-30

**Authors:** Xiang Gao, Wen-Hui Yan, Bo-Yang Hu, Yu-Xin Huang, Shi-Mei Zheng

**Affiliations:** College of Chemistry and Chemical Engineering, Weifang University, Weifang 261061, China

**Keywords:** MOF, light hydrocarbon, gas separation

## Abstract

The separation of light hydrocarbon compounds is an important process in the chemical industry. Currently, its separation methods mainly include distillation, membrane separation, and physical adsorption. However, these traditional methods or materials have some drawbacks and disadvantages, such as expensive equipment costs and high energy consumption, poor selectivity, low separation ratios, and separation efficiencies. Therefore, it is important to develop novel separation materials for light hydrocarbon separation. As a new type of organic–inorganic hybrid crystalline material, metal–organic frameworks (MOFs) are promising materials for light hydrocarbon separation due to their designability of structure and easy modulation of function. This review provides an overview of recent advances in the design, synthesis, and application of MOFs for light hydrocarbon separation in recent years, with a focus on the separation of alkane, alkene, and alkyne. We discuss strategies for improving the adsorption selectivity and capacity of MOFs, including pore size limitation, physical adsorption, and chemisorption. In addition, we discuss the advantages/disadvantages, challenges, and prospects of MOFs in the separation of light hydrocarbon.

## 1. Introduction

Light hydrocarbon separation is one of the important processes in the chemical industry [1,2,3,4,5,6]. The separated and purified light hydrocarbon compounds are important energy fuels and chemical raw materials, which are widely used in the preparation of synthetic fibers, synthetic rubbers, synthetic plastics, and organic compounds. However, traditional light hydrocarbon separation materials and methods (physical adsorption, membrane separation, distillation, etc.) have some disadvantages. For example, traditional physical adsorption materials lack weak interaction sites with light hydrocarbon compounds and have a limited specific surface area, resulting in a limited selectivity and adsorption capacity for light hydrocarbons, which leads to poor separation. Membrane separation materials solve the shortcomings of physical adsorption materials to a certain extent, but their gas permeability is not good, which leads to the poor efficiency of the separation of light hydrocarbons. The distillation process is based on the different boiling points of different components in the mixture to realize the effective separation of light hydrocarbons, but its disadvantage lies in the need for expensive and bulky distillation equipment, as well as the problems of high energy consumption and high pollution. Therefore, to efficiently separate light hydrocarbons and utilize resources, reduce environmental pollution, and achieve sustainable development, it is urgent to develop new materials or methods for the separation of light hydrocarbons.

**Figure 1:**
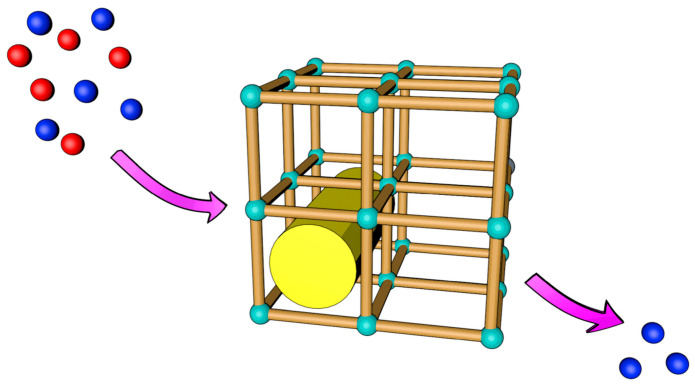
Graphical abstract of the metal–organic framework for gas separation.

As a new type of organic–inorganic hybridized porous crystalline material, metal–organic frameworks (MOFs), with the features of a designable structure and easily adjustable nature, have shown good application prospects in many fields such as gas storage and separation [7,8,9], optics [10,11,12], fluorescence sensing [13,14,15], catalysis [16,17,18], magnetism [19,20,21], biomedicine [22,23,24,25], etc. [26,27]. As materials with porous structures, MOFs show great potential in the field of light hydrocarbon separation [28,29,30,31,32,33]. MOFs are crystalline materials self-assembled by organic ligands and metal ions through coordination bonds, with a regular structure, a certain specific surface area, an adjustable pore size, and various methods of functionalization modification. These characteristics enable MOFs to optimize and enhance their ability regarding the separation of light hydrocarbons through pore size adjustment, increasing adsorption sites, and enhancing the interaction force with light hydrocarbon molecules. In addition, the diversity of MOFs species, the simplicity of the synthesis method, the mildness of the synthesis conditions, and the relative structural robustness of the MOFs further make them strong candidates for new light hydrocarbon separation materials (Figure 1).

In this review, the applications and progress of MOFs in light hydrocarbon separation are presented. First, the current research statuses of MOFs in the separation of light hydrocarbon-containing mixtures (such as the alkane-containing mixture, alkene-containing mixture, alkyne-containing mixture, C_4_ hydrocarbon mixture, and C_6_ isomers mixture) are introduced, which can help readers to understand the connection between MOFs and light hydrocarbon separation. Secondly, the mechanism of MOFs in the separation of light hydrocarbons is elucidated, the factors affecting the separation performance of MOFs are summarized, and the strategies for enhancing the separation performance of MOFs in the separation of light hydrocarbons are explored. Finally, the challenges faced by MOFs and the use of MOFs in the field of light hydrocarbon separation are summarized and prospected, respectively. This review can help the readers to construct the knowledge network of separating light hydrocarbon using MOFs.

## 2. Porous MOFs for Light Hydrocarbon Separation

The structural versatility, designability, and functional tunability of porous MOF materials allow them to be applied in the field of gas separation. Thanks to the efforts of researchers, MOFs have been widely used in the separation of light hydrocarbon mixtures. In the next section, we will summarize the research of MOF materials for the separation of CH_4_/CO_2_, CH_4_/N_2_, C_2_H_2_/C_2_H_4_, C_2_H_2_/CO_2_, C_3_H_6_/C_3_H_8_, C_4_ hydrocarbons, and C_6_ isomers mixtures (Table 1).

### 2.1. CH_4_/N_2_ and CH_4_/CO_2_ Separation

Methane (CH_4_) is a colorless, odorless gas, mainly composed of carbon and hydrogen. There are two main sources of CH_4_, one is underground natural gas, and CH_4_ is usually mixed with other hydrocarbons; the other is produced through the fermentation and decomposition process of microorganisms in biomass (e.g., sludge, biogas, compost, etc.). CH_4_ is an important energy fuel, which can not only be burned directly but can also be compressed and liquefied as a source of power for new energy vehicles. At the same time, CH_4_ is an important raw material in the chemical industry and can be converted into other chemical products [34,35,36]. In addition, CH_4_ has the advantages of high combustion efficiency and relatively harmless combustion products. However, unpurified CH_4_ often contains nitrogen (N_2_) and carbon dioxide (CO_2_), which greatly reduce the combustion heat value of CH_4_ and affect the preparation of CH_4_-based chemical products, so it is of great significance to separate CH_4_ from N_2_ and CO_2_. As a new type of separating material, MOFs show great potential in the field of CH_4_/N_2_ and CH_4_/CO_2_ separation.

The separation of CH_4_/N_2_ can be achieved more efficiently based on a thermodynamic–kinetic synergistic separation mechanism than on a single thermodynamic or kinetic separation mechanism. In 2022, Zhang et al. [37] chose aluminum nitrate and 1,2,4,5-benzenetetracarboxylic acid (BTEC) as the main raw materials for the synthesis of MIL-120Al. MIL-120Al has rhombic 1D nonpolar pores formed by benzene rings (parallel to the channel direction), and its pore size (0.54 × 0.47 nm) is comparable to the kinetic diameter of methane molecules, which is capable of the synergistic thermodynamic–kinetic separation of the CH_4_/N_2_ mixture. The specific surface area of MIL-120Al is 529 m^2^/g. Single-component CH_4_ and N_2_ gas adsorption experiments show that the adsorption capacity of MIL-120Al for CH_4_ is 33.7 cm^3^/g (298 K, 1 bar), which is the highest among reported aluminum-based MOFs. IAST calculations show that the selectivity of MIL-120Al for an equimolar CH_4_/N_2_ mixture is 6.0 at 298 K and 1 bar, which is much higher than that of most reported MOFs (e.g., ZIF-8: 2.5, NU-1000: 2.5, MOF-177: 4.0, MIL-53-Al: 3.7, Uio-66-Br_2_: 5.1). The GCMC simulation results show that the pores of MIL-120Al are the preferential adsorption sites for CH_4_ and N_2_ and that the distribution probability of CH_4_ is much higher than that of N_2_. The breakthrough experiments further demonstrate that MIL-120Al can completely realize the separation of the CH_4_/N_2_ mixture, and the separation performance is not affected by the presence of H_2_O. Pressure swing adsorption (PSA) process simulations show that MIL-120Al can enrich the original 50% methane to 86% for a mixed CH_4_/N_2_ (50/50, *v*/*v*) mixture.

The dense open-metal sites and exposed pyridine N-atom sites can generate weak non-bonding interactions with CO_2_ molecules, which contribute to the enhancement of the separation ability of MOFs for CH_4_/CO_2_. In 2022, a novel 2D Co-MOF based on a nicotinic acid-based organic ligand was synthesized by Qin et al. [38]. Single X-ray crystal diffraction shows that the 2D layers in the Co-MOF are interconnected through hydrogen bonding and pi–pi stacking to form a porous 3D framework with channels. Since hydrogen bonding and pi–pi stacking are weak interactions, these 2D layers have a certain sliding property under external stimulation, which makes the extended 3D framework have the characteristics of a dynamic MOF. Gas adsorption experiments show that the adsorption capacities of the activated Co-MOF on different gases are in the order of CO_2_ > C_2_H_2_ > CH_4_ > N_2_ > CO. Among them, the adsorption curve of CO_2_ shows a sharp increase at low pressures, which indicates that there is a strong interaction between the activated Co-MOF and CO_2_. The maximum uptake of activated Co-MOF for CO_2_ is 9.77 wt% (2.22 mmol/g) at 273 K and 1 bar. The mechanism is further investigated by in situ DRIFTS analysis. The typical asymmetric CO_2_ stretching vibration band is observed at 2200 ~ 2400 cm^−1^, and the bands at 1727 and 1587 cm^−1^ could be attributed to COOH or HCOO^-^. The results indicate that the adsorbed CO_2_ molecules are converted to COOH or HCOO^-^ by the open-metal sites of CO^2+^. IAST calculations showed that the selectivity of the activated Co-MOF for CH_4_/CO_2_ (50/50, *v*/*v*) was 37.2 (298 K, 1 bar), which is comparable to that of reported 2D/3D MOFs. GCMC simulations and DFT calculations show that the appropriate pore size, thickness of the layer structure, dense pyridine N-atom in the inner wall of the channel, and open-metal sites are favorable for the selective capture of CO_2_.

### 2.2. C_2_H_2_/C_2_H_4_ Separation

Acetylene (C_2_H_2_) and ethylene (C_2_H_4_) are the simplest alkynes and olefins, which originate from the cleavage and recombination of higher hydrocarbons and usually exist in the form of mixtures. C_2_H_2_ and C_2_H_4_ play important roles in human daily life and industry [39,40,41]. C_2_H_2_ can be used as a high-temperature fuel, and it has a wide range of applications in the field of metal cutting and gas welding. C_2_H_4_ is the main raw material for the manufacture of polyethylene (PE), which is a common plastic used in the packaging and manufacturing industries. C_2_H_2_ and C_2_H_4_ are important chemical raw materials, which can be used for synthetic rubbers, synthetic fibers, and other polymers. C_2_H_2_ and C_2_H_4_ also play important roles in the field of bioregulation. C_2_H_2_ can be used as a plant growth regulator for the promotion of plant growth and the ripening of fruit, and C_2_H_4_ can be used for the preservation of fruits and vegetables. In addition, it is necessary to separate C_2_H_2_ and C_2_H_4_ because acetylene impurities within olefins can lead to the poisoning of catalysts used in olefin polymerization reactions, resulting in lower polymerization reaction efficiencies and yields. However, due to the similarity of C_2_H_2_ and C_2_H_4_ molecules in terms of their physicochemical properties (e.g., molecular volume, existential morphology, density, boiling point, and structure), it is difficult to achieve the efficient and selective separation of the two by existing methods and materials (e.g., adsorptive separation based on molecular sieve materials, membrane separation and separation by chemical reaction, etc.). Therefore, realizing the effective separation of C_2_H_2_ and C_2_H_4_ has become a very challenging research topic. Thanks to the characteristics of the structure and properties of porous MOF materials, they can realize the effective separation of C_2_H_2_ and C_2_H_4_ with a low energy consumption, high efficiency, and high selectivity.

Introducing open-metal sites into MOFs with narrow channels is an effective method for achieving a high C_2_H_2_/C_2_H_4_ separation ratio. In 2023, Pal et al. [42] used cadmium nitrate, thiophene-2,5-dicarboxylic acid (H_2_TDC), and 1,2-bis(1-(pyridin-3-yl)ethylidene)hydrazine as raw materials and constructed a 3D MOF (IITKGP-30) with 1D narrow pores. Single-crystal X-ray diffraction experiment shows that in IITKGP-30, H_2_TDC ligands and cadmium ions formed two-dimensional layers, and the flexible ligands acted as pillars. IITKGP-30 has a *rob*-type topology, and the diameter of its internal 1D aperture is about 0.45 × 0.45 nm (in the direction of the crystallographic c-axis). The solvent-accessible void of IITKGP-30 is 19.7%, computed by the PLATON software. The IAST selectivity (295 K, 1 bar) of IITKGP-30 for C_2_H_2_/C_2_H_4_ (1:99, 50:50, *v*/*v*) was 3.3 and 6.2, respectively, which are greater than the values for NOTT-300 (2.17), Fe-MOF-74 (2.08), M’MOF-2a (1.93), and UiO-67(NH_2_)_2_ (2.1). Theoretical calculations suggest that the C_2_H_2_ molecules will preferentially interact with the exposed metal sites in IITKGP-30 via an end-to-end mode. Breakthrough experiments were carried out at 298 K; the results showed that the C_2_H_2_/C_2_H_4_ (1:99, *v*/*v*) mixture achieved complete separation with a penetration selectivity of 2.4. In addition, IITKGP-30 has superb water stability and can maintain its separation performance under a humid environment (relative humidity: 97%) after at least 15 days.

Apart from the open-metal sites, other weak interactions (e.g., hydrogen-bond interaction and pi interaction) can also facilitate the selective adsorption of C_2_H_2_ over C_2_H_4_. Recently, Huang et al. [43] used low-cost ligands and Ni salts as raw materials and synthesized a stable microporous Ni-MOF(TJE-1). TJE-1 has 1D channels with a pore size of 0.52 × 0.52 nm. The specific surface area of activated TJE-1 is 827 m^2^ g^−1^. At 298 K and 1 bar, the adsorption capacity of TJE-1 is 117 mL g^−1^ (5.27 mmol g^−1^) for C_2_H_2_ and 69 mL g^−1^ (3.08 mmol g^−1^) for C_2_H_4_. The adsorption capacity of TJE-1 for C_2_H_2_ is comparable to that of many well-known MOFs materials, such as NKMOF-1-Ni (2.72 mmol g^−1^), UTSA-100 (4.27 mmol g^−1^), NOTT-300 (6.34 mmol g^−1^), and Fe-MOF-74 (6.8 mmol g^−1^). The Q*_st_* values of TJE-1 for C_2_H_2_ and C_2_H_4_ were 35.5 and 29.9 kJ mol^−1^, indicating that TJE-1 has stronger adsorption for C_2_H_2_. In addition, TJE-1 has excellent stability and can maintain its crystalline structure when exposed to an acid/base solution, hot water, and common organic solvents. Breakthrough experiments show that TJE-1 has a separation factor of 2.25 for an equimolar C_2_H_2_/C_2_H_4_ mixture, realizing the efficient separation of C_2_H_2_/C_2_H_4_. Theoretical calculations showed that hydrogen bonds and pi interactions were formed between the TJE-1 framework and the C_2_H_2_ molecules. This study guides the development of next-generation MOF materials for industrial separation (green and economical raw materials, convenient preparation means, and good separation results), which is of great significance for industrial gas separation.

### 2.3. C_2_H_2_/CO_2_ Separation

The separation of C_2_H_2_/CO_2_ is more intractable than the separation of C_2_H_2_/C_2_H_4_. The CO_2_ component in C_2_H_2_ usually comes from the cracking reaction of C_2_H_4_, and the small number of CO_2_ impurities contained in the feed gas will be transmitted to the C_2_H_2_ product [44,45,46]. Compared with C_2_H_2_ and C_2_H_4_, C_2_H_2_ and CO_2_ have a higher similarity in physicochemical properties. They are both linear molecules with boiling points of 188.40 K and 216.55 K, critical temperatures of 308.3 K and 304.12 K, critical pressures of 61.14 bar and 73.74 bar, and molecular dynamics diameters of 0.33 nm. The dipole moments are both 0, and the polarizabilities are 33.3~39.3 × 10^25^/cm^3^ and 29.11 × 10^25^/cm^3^, respectively. The extreme similarity of the physicochemical properties creates difficulties in separating the gas mixture. How to realize the effective separation of C_2_H_2_/CO_2_ has become a major problem in front of researchers. With the deepening of the research on MOF materials, researchers have found that MOF materials can realize the low-energy and high-efficiency separation of C_2_H_2_/CO_2_, and MOF materials have shown the incomparable advantages of traditional methods/materials and the potential application in the separation of the C_2_H_2_/CO_2_ mixture.

**Figure 2:**
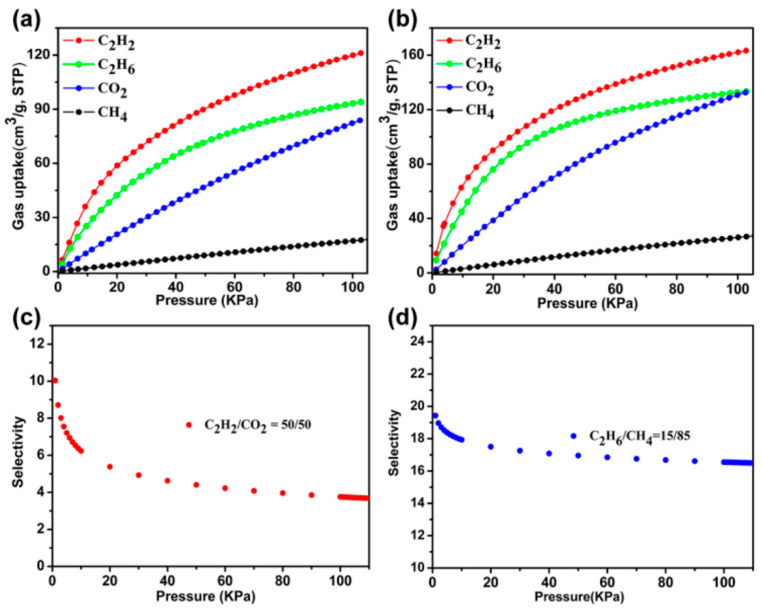
(**a**,**b**) Gas adsorption isotherms of Cu-MOF at 298/273 K. (**c**,**d**) The IAST selectivity of C_2_H_2_/CO_2_ (50/50, 298 K) and C_2_H_6_/CH_4_ (15/85, 298 K). Reprinted with permission from Ref. [44]. Copyright 2021, American Chemical Society.

The kind, quantity, and location of functional groups belonging to the organic ligand in the MOF structures play an important role in the improvement of the C_2_H_2_/CO_2_ gas separation performance. In 2021, Li et al. [47] constructed an MOF based on the classical double Cu-paddlewheel structure as secondary building units (SBUs) with di-isophthalic acid ligands (functional groups: -NH_2_ and -OCF_3_) for C_2_H_2_/CO_2_ separation. Single X-ray crystal diffraction analysis demonstrates the presence of three types of cages (cages A, B, and C) in Cu-MOF, which are connected to each other to form interspersed channels. The PLATON calculation result shows that the solvent-accessible volume of Cu-MOF is about 58.4%. Thermogravimetric analysis shows that the Cu-MOF can still maintain its original crystalline structure at 300 °C. The N_2_ adsorption and desorption curves show that the saturated adsorption capacity of the activated Cu-MOF for N_2_ is 430 cm^3^/g, and its BET surface area is 1617 m^2^/g. Gas adsorption experiments on C_2_H_2_ and CO_2_ show that the adsorption capacity of Cu-MOF for C_2_H_2_ is higher than that of CO_2_. The calculated Q*_st_* value of C_2_H_2_ is significantly higher than that of CO_2_, indicating that Cu-MOF has stronger adsorption for C_2_H_2_. The selectivity was calculated by ideal adsorption solution theory (IAST) at 298 K for the mixed C_2_H_2_/CO_2_ (50/50) fractions, which ranged from 3.68 to 10.03 (Figure 2).

**Figure 3:**
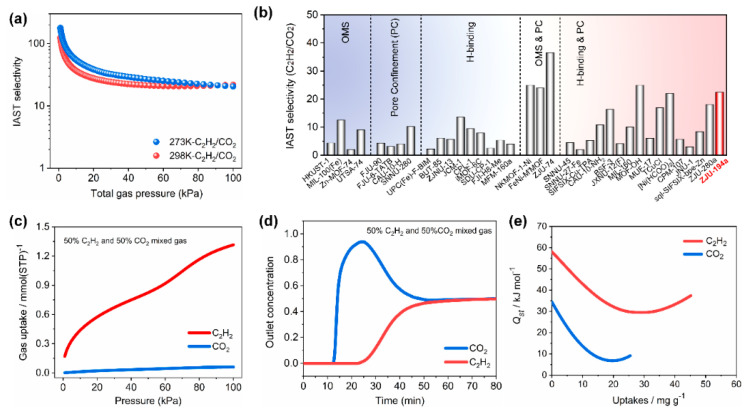
The IAST selectivity (**a**,**b**), dynamic breakthrough curves (**c**,**d**), and isosteric heat (**e**) of ZJU−194a towards the C_2_H_2_/CO_2_ (50/50, *v*/*v*) gas mixture. Reprinted with permission from Ref. [45]. Copyright 2023, Elsevier B.V.

In contrast to the traditional adsorption mechanism of guest molecules by MOFs, the novel gate-breathing (gate-opening and -closing) effect can reach unpredictable C_2_H_2_/CO_2_ separation results. Not long ago, Jiang et al. [48] used copper salt (copper perchlorate) and imidazole-derived dmpbi (1,1′-(2,5-dimethyl-1,4-phenylene)bis(1 H-imidazole)) as an organic ligand, successfully constructed a novel perchlorate-based microporous MOF(ZJU−194), and applied it to the separation of a C_2_H_2_/CO_2_ mixture. The inner wall of ZJU−194 pores is enriched with bare oxygen atoms, which can generate strong interaction forces with the guest molecules. ZJU−194 can retain its structure not only in water (submerged in water for a day) but also at high temperatures (below 305 °C), which exhibits a good chemical/thermal stability nature. Gas adsorption tests showed that the activated ZJU−194a exhibits a two-step door-opening effect for C_2_H_2_ and a blocking effect for CO_2_, i.e., ZJU−194a allows for the passage of C_2_H_2_ molecules but prevents the passage of CO_2_ molecules. The adsorption heat (Q*_st_*) values of ZJU−194a for C_2_H_2_ and CO_2_ are 58.1 kJ/mol and 34.4 kJ/mol, respectively. The selectivity of ZJU−194a for C_2_H_2_/CO_2_ (50/50, *v*/*v*) is as high as 22.4, which is much higher than that of most of the reported MOF materials (including MOFs with high-density open-metal sites). Dynamic breakthrough experiments show that ZJU−194a can realize the complete separation of C_2_H_2_/CO_2_, which further validates the excellent performance of ZJU−194a (Figure 3).

### 2.4. C_3_H_6_/C_3_H_8_ Separation

Propylene (C_3_H_6_) has an important role in the petrochemical field, not only for the production of a variety of high-value-added organic chemicals (acrylic acid, acrolein, etc.) but also as the main raw material for the preparation of organic polymers (polypropylene, synthetic rubber). Among them, polypropylene (PP) with a high strength, heat resistance and chemical stability, and other advantages is widely used in packaging materials, plastic products, automotive parts, and so on. Polypropylene synthetic rubber has excellent elasticity, abrasion resistance, and chemical resistance and is used in automobile tires and pipeline manufacturing, etc. As is well known, C_3_H_6_ is mainly produced through the cracking of propane (C_3_H_8_) or naphtha and is purified by utilizing distillation and membrane separation [49,50,51]. However, due to the similarity between C_3_H_6_ and C_3_H_8_ in terms of physicochemical properties (boiling point, polarity, molecular size, etc.), the above methods consume a large amount of energy in C_3_H_6_/C_3_H_8_ separation and cause large pollution to the environment, which is not in line with the concept of building a resource-saving and environment-friendly society. Therefore, there is an urgent need to develop a novel material or method for C_3_H_6_/C_3_H_8_ separation. Currently, porous MOFs have been demonstrated to be used for the efficient and economical separation of the C_3_H_6_/C_3_H_8_ mixture.

**Figure 4:**
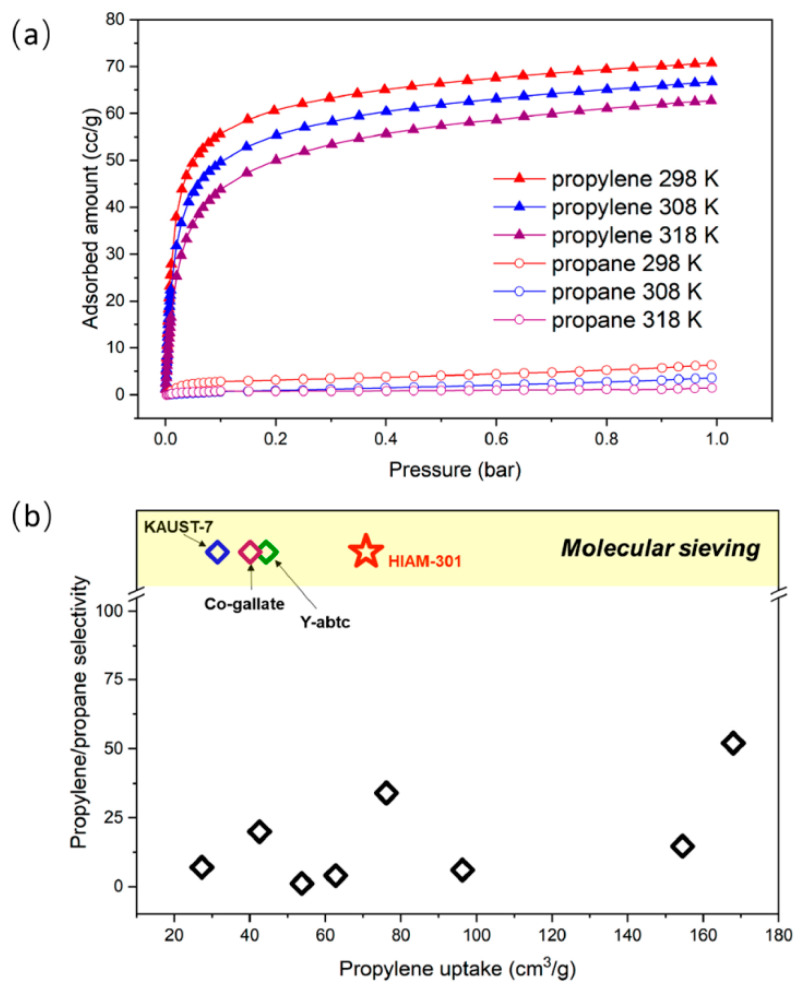
(**a**) Adsorption isotherms of C_3_H_6_ and C_3_H_8_ (298 K, 308 K and 318 K) and (**b**) C_3_H_6_ and C_3_H_8_ selectivity comparison chart. Reprinted with permission from Ref. [49]. Copyright 2021, American Chemical Society.

The pore size of MOFs can allow or prevent the passage of specific molecules (the sieving effect of MOF) [52]. Based on this property of MOFs, it is possible to apply it to the separation of gas mixtures and to obtain high-purity single-component gas. In 2021, Yu et al. [53] designed a Y-based MOF material, Y_6_(OH)_8_(eddi)_3_(DMA)_2_ (HIAM-301). HIAM-301 has an *ftw*-type topology with highly contracted and distorted cubic cages and pore diameters, which creates a peculiarly aberrant configuration. Due to the active diffusion caused by the narrow pore size, HIAM-301 exhibits a low adsorption of N_2_ at 77 K. The specific surface area of HIAM-301 was successfully obtained (579 m^2^/g) using CO_2_ as the probe molecule at 195 K. At 298 K and 1 bar, HIAM-301 exhibits an obvious pore-sieving effect on C_3_H_6_ and C_3_H_8_, with the C_3_H_6_ adsorption capacity of 3.16 mmol/g and molecular exclusion of C_3_H_8_ (<0.3 mmol/g) (Figure 4). IAST calculations show that the selectivity of HIAM-301 for the C_3_H_6_/C_3_H_8_ mixture is as high as 150. The Q*_st_* of HIAM-301 for C_3_H_6_ was about 27 kJ/mol, which is higher than that of JNU-3a (benchmark) and MOFs with open metal sites (KAUST-7, Y-abtc, and Co-gallate). The low Q*_st_* is favorable for the energy-saving desorption of C_3_H_6_ molecules by HIAM-301 in practical applications. The results of breakthrough experiments show that C_3_H_8_ is eluted at the beginning, while C_3_H_6_ can be retained in the column for a longer time (∼35 min/g). Notably, HIAM-301 can produce polymerization-grade C_3_H_6_ feedstocks (with a purity as high as 99.6%). In situ neutron powder diffraction and inelastic neutron scattering demonstrated the sites of C_3_H_6_ molecules in HIAM-301 and the molecular dynamics of host–guest interaction (strong hydrogen bond and pi–pi stacking interaction). This study provides a new benchmark for the separation of C_3_H_6_/C_3_H_8_ at that time.

**Figure 5:**
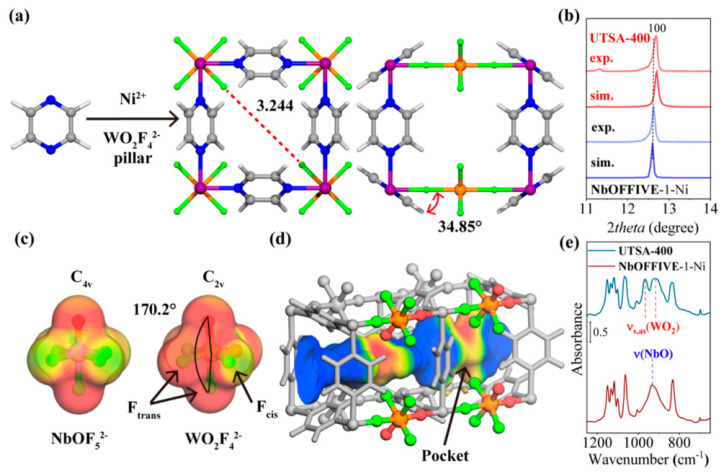
(**a**−**d**) Structure, PXRD, molecular electrostatic potential, connolly surface (mapped with electrostatic potential) of UTSA−400, and (**e**) partial IR spectra of UTSA−400. Reprinted with permission from Ref. [50]. Copyright 2023, American Chemical Society.

The functional groups (e.g., -NH_2_, -COOH, and -F) and exposed Lewis basic sites (e.g., O, N) in the structure of porous MOFs are capable of forming multiple hydrogen bonds or van der Waals interactions with the guest molecules, and this synergistic mechanism can dramatically enhance the separation ability of MOFs for the C_3_H_6_/C_3_H_8_ mixture. In 2022, Xie et al. [54] used NbOFFIVE-1-Ni as a blueprint and prepared a stable oxyfluoride-based ultra-microporous MOF (UTSA−400) by replacing the anionic part (NbOF_5_^2−^) in the original structure with WO_2_F_4_^2−^. Single X-ray crystal diffraction shows that the pyrazine rings and WO_2_F_4_^2−^ part in the structure of UTSA−400 are disordered, which resulted in the shrinkage of the pore volume in UTSA-400 (Figure 5). Calculations show that the O- and F-atom sites of WO_2_F_4_^2−^ parts have a more negative molecular electrostatic potential compared to NbOF_5_^2−^, implying that it can generate a stronger force with the guest molecule. UTSA−400 has a specific surface area of 226 m^2^/g with a high adsorption of 46.3 cm^3^·cm^−3^ for C_3_H_6_ at a low pressure (0.1 bar) and a high adsorption capacity of 92.1 cm^3^·cm^−3^ for C_3_H_6_ at 1 bar. Compared to NbOFFIVE-1-Ni, the C_3_H_6_ capacity of UTSA−400 is significantly improved by 544% and 69%, which is higher than that of the common zeolites and most of the reported C_3_H_6_/C_3_H_8_-separation MOFs. The IAST selectivity of UTSA−400 for an equimolar C_3_H_6_/C_3_H_8_ mixture fraction is more than 107 at 298 K and 1 bar, exceeding all other reported MOFs (including those with molecular screening effects), and the Q*_st_* of C_3_H_6_ to UTSA−400 is 60.5 kJ/mol. In situ infrared spectroscopy characterization demonstrated that the CH/CH_2_ group of C_3_H_6_ exhibits different states in NbOFFIVE-1-Ni and UTSA−400, which is related to the NbOF_5_^2−^ and WO_2_F_4_^2−^ anions. First-principles dispersion-corrected DFT (DFT-D) calculations show that not only does C_3_H_6_ form hydrogen-bonding interactions with the bare O- and F-atom sites belonging to the WO_2_F_4_^2−^ anions, but the vinyl group in C_3_H_6_ also forms C-H^...^pi van der Waals interactions with pyrazine rings.

### 2.5. C_4_ Hydrocarbon and C_6_ Isomers Separation

In recent years, the separation of C_4_ and C_6_ hydrocarbons has attracted increasing attention. Unlike the aforementioned C_1_/C_2_/C_3_ hydrocarbons separation, the separation of C_4_ and C_6_ hydrocarbons has focused more on the isomer’s separation. C_4_ hydrocarbons are not only a kind of clean fuel but also a raw material for the synthesis of other chemicals (e.g., polymers, synthetic rubbers, and resins) [55,56,57]. C_4_ hydrocarbons occupy a certain market size; thus, obtaining high-purity C_4_ hydrocarbons is of great importance. Currently, C_4_ hydrocarbons have been studied mainly for the separation of butane isomers (*n*-C_4_H_10_ and *i*-C_4_H_10_) and butene isomers (*n*-butene and *i*-butene, *cis*-2-butene, and *trans*-2-butene). The study of C_6_ hydrocarbons is mainly for the separation of hexane (C_6_H_14_) isomers. C_6_H_14_ has a variety of different molecular configurations (including chiral structures), and these isomers are extremely similar in terms of physical properties (e.g., boiling points, densities), which makes it difficult to achieve the effective separation of them by conventional physical separation methods (e.g., distillation, membrane separation) [58,59,60]. In addition, the minimal differences in chemical properties between the C_6_H_14_ isomers also make it difficult to selectively transform the isomers by chemical reactions. Obviously, the separation of C_4_ and C_6_ hydrocarbons is more complicated, and to obtain products with a higher market value, there is an urgent need to develop new materials or methods to realize the effective separation of these hydrocarbons. Fortunately, MOFs can solve this urgent problem to a certain extent.

**Figure 6:**
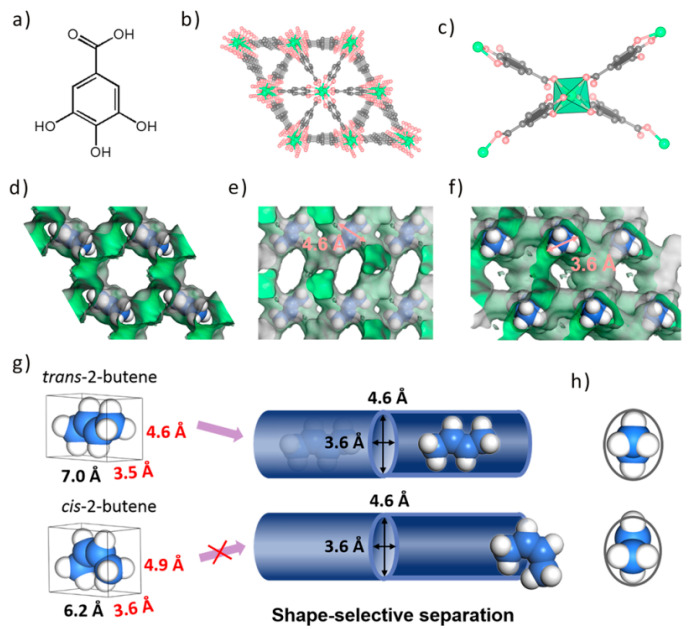
(**a**–**f**) Structure of M-gallate and (**g**,**h**) schematic illustration of the shape-selective separation mechanism. Reprinted with permission from Ref. [57]. Copyright 2020, American Chemical Society.

Based on the size/shape-selective separation effect and van der Waals weak interaction, MOFs can accurately recognize the tiny differences between the C_4_ isomers at the atomic level and realize the effective separation of C_4_ isomers. In 2020, Chen et al. [61] constructed a series of isostructural MOFs (M-gallate, M = Ni, Mg, Co) under solvothermal conditions based on gallic acid with nickel, magnesium, and cobalt salts, respectively. Single X-ray crystal diffraction characterization proved that the M-gallate has elliptical pore channels (the pore size of Mg-gallate is 0.36 × 0.46 nm). Among the M-gallate, Mg-gallate shows the best separation performance for C_4_ hydrocarbon isomers due to the pore size obstruction, which prevented *cis*-2-butene from entering into the pore channels of Mg-gallate (Figure 6). Single-component adsorption curves show that the IAST selectivity of Mg-gallate for *cis*/*trans*-2-butene is 3.2 at 298 K and 1.0 bar, which surpassed the benchmarks of RUB- 41 (1.2), clinoptilolite (1.6), ZJNU-30a (1.1), and Fe-MOF-74 (1.0). DFT calculations show that strong C^...^H-O synergistic supramolecular interactions are formed between Mg-gallate and the C_4_ hydrocarbon isomer. Breakthrough experiments show that Mg-gallate could not only separate the two-component *trans*/*cis*-2-butene and 1-butene/*iso*-butene but also realize the effective separation of the three-component 1,3-butadiene/1-butene/*iso*-butene.

**Figure 7:**
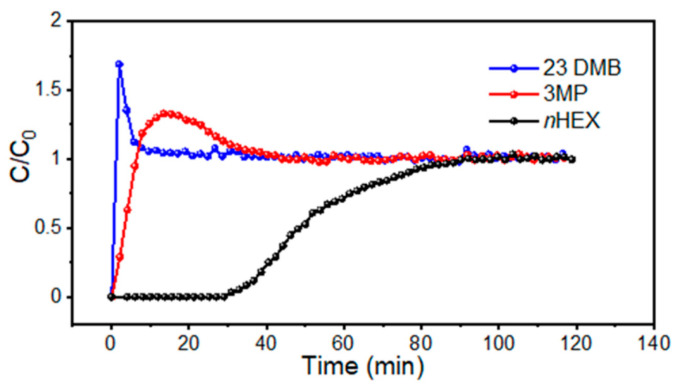
Breakthrough curves of *n*HEX/3MP/23DMB (equimolar ternary mixture) at 303.15 K. Reprinted with permission from Ref. [58]. Copyright 2021, American Chemical Society.

Compared to rigid MOFs, flexible MOFs exhibit a higher structural flexibility, tunability, and adaptability, which endows them with unpredictable properties and greater potential in the field of gas separation. In 2021, Velasco et al. [62] reported a novel flexible Zn-MOF (Zn-adtb) with a rare (4,8)-connected *scu* topology. Based on kinetic and molecular sieving mechanisms, Zn-adtb can realize efficient C_6_ isomers separation (i.e., the linear n-hexane, *n*HEX; single-branched 3-methylpentane, 3MP; dibranched 2,3 dimethylbutane, 23DMB). The activated Zn-adtb exhibited different adsorption behaviors for C_6_ isomers with different branched chains. Zn-adtb shows a high adsorption capacity for the linear isomer *n*HEX, while the adsorption of the single-branched isomer 3MP and the dibranched isomer 23DMB is almost negligible. Breakthrough experiments further demonstrated the ability of Zn-adtb to completely separate the linear C_6_ isomers from the branched isomers. The branched isomers 23DMB and 3MP are successively and rapidly eluted from the Zn-adtb-populated column, whereas the retention time of the linear isomer *n*HEX within the packed column is as high as 30 min (Figure 7). In addition, solid-state NMR (ssNMR), in situ FTIR analysis, and first-principles density functional theory calculations further revealed the mechanism of C_6_ isomers separation, and the results showed that the separation of *n*HEX/3MP and *n*HEX/23DMB belonged to different mechanisms; the former is a kinetic separation mechanism and the latter is a molecular sieving mechanism.

## 3. Mechanisms and Strategies for Improving the MOFs Separation Ability

From the above description, it can be summarized that there are several mechanisms for the separation of light hydrocarbons by MOFs:

(1) Pore size/shape sieving by rigid MOFs. Based on the degree of match between the size/shape of the guest molecule and the pore size/shape of the MOFs, the guest molecule is selectively allowed to pass through.
(2) Breathing effect (gate effect) of flexible MOFs. Under specific external stimuli (e.g., temperature, pressure, gas, etc.), the structure of MOFs changes to allow or prevent the passage of guest molecules.
(3) Hydrogen bonding, van der Waals forces, and exposed electron-rich sites. Hydrogen bonds (e.g., C-H^...^F, C-H^...^O, C-H^...^pi, etc.), van der Waals forces, and exposed electron-rich sites (N- or O-atom, etc.) play key roles in the gas separation process, and they endow the weak interactions between the MOF frameworks and the guest molecules
(4) Open metal sites. Open metal sites can form strong non-bonding interactions with the guest molecules, and sometimes they can even break through the limit and form chemical bonds with the guest molecules.

To further enhance the selective separation performance of MOFs for light hydrocarbons, MOFs can be optimized in terms of the following aspects:

(1) Selecting organic ligands with compact sizes as raw materials to obtain MOFs with microporous structures.
(2) Designing, constructing, and optimizing the structure of MOFs based on secondary building blocks and topology. For example, introducing open metal sites/functional groups (e.g., -NH_2_, -OH, -CF_3_, etc.) into the structure of MOFs not only regulates the pore size/shape of MOFs but also increases the possibility of forming weak interactions between MOFs and guest molecules.
(3) Maximizing the selectivity of MOFs for light hydrocarbons through the synergistic effect of multiple mechanisms.

## 4. Conclusions and Prospects

In recent decades, MOFs have been developed considerably, and many classical and well-known MOFs have been reported, such as MOF-5, ZIF-8, HKUST-1, UiO-66, MIL-101, MOF-177, IRMOF-1, and NU-1000. Based on the mechanisms of pore size sieving, breathing effects (gate effect), physical adsorption (non-bonding weak interactions), and chemical adsorption (guest molecule bonding and transformation), MOFs can realize the effective separation of mixed components of alkanes, olefins, and alkynes. The separation benchmarks of MOFs toward gas mixtures have been broken continuously, showing excellent separation performance and application prospects of MOFs in the field of light hydrocarbon separation. The advantages of MOFs (a regular structure, a certain, specific surface area, an adjustable pore size, various methods of functionalization modification, etc.) far outweigh those of conventional materials and methods, which indicates that MOFs can be used as a new type of material for the separation of light hydrocarbons. However, MOFs also have many problems to be improved and solved, such as reducing the cost of MOFs synthesis, the large-scale preparation of MOFs, improving the stability of MOFs, the reduced generation of pollutants during MOF synthesis, the preparation of environment-friendly MOFs, realizing the long-cycle recycling of MOFs, etc. It is believed that with the continuous efforts of researchers and the deepening research on MOFs, these problems will be solved, and it is foreseeable that the application of MOFs materials in the field of light hydrocarbon separation will be more diverse and extensive in the future.

## Figures and Tables

**Table 1:** Kinetic diameters of selected molecules.

Adsorbate	Kinetic Diameter/nm	Adsorbate	Kinetic Diameter/nm
N_2_	0.364–0.380	*n*-C_4_H_10_	0.4687
CO_2_	0.33	*i*-C_4_H_10_	0.5278
CH_4_	0.375	1-Butene	0.45
C_2_H_2_	0.33	*cis*-2-butene	0.423
C_2_H_4_	0.4163	1,3-Butadiene	0.52
C_3_H_6_	0.4678	*n*-C_6_H_14_	0.43
C_3_H_8_	0.43–0.5118		

## Data Availability

Not applicable.
